# Cytotoxic and Inflammatory Potential of Air Samples from Occupational Settings with Exposure to Organic Dust

**DOI:** 10.3390/toxics5010008

**Published:** 2017-03-01

**Authors:** Susana Viegas, Liliana Aranha Caetano, Merja Korkalainen, Tiago Faria, Cátia Pacífico, Elisabete Carolino, Anita Quintal Gomes, Carla Viegas

**Affiliations:** 1 Environment and Health Research Group, Escola Superior de Tecnologia da Saúde de Lisboa, ESTeSL, Instituto Politécnico de Lisboa, Av. D. João II, Lote 4.69.01, 1990-096 Lisboa, Portugal; Liliana.caetano@estesl.ipl.pt (L.A.C.); tiagof_scp@hotmail.com (T.F.); catia.s.pacifico@gmail.com (C.P.); etcarolino@estesl.ipl.pt (E.C.); anita.gomes@estesl.ipl.pt (A.Q.G.); carla.viegas@estesl.ipl.pt (C.V.); 2 Centro de Investigação em Saúde Pública, Escola Nacional de Saúde Pública, Universidade NOVA de Lisboa, 1600-560 Lisbon, Portugal; 3 Research Institute for Medicines (iMed.ULisboa), Faculty of Pharmacy, University of Lisbon, 649-003 Lisbon, Portugal; 4 National Institute for Health and Welfare (THL), Department of Health Security, Chemicals and Health Unit, P.O. Box 95, FIN-70701 Kuopio, Finland; merja.korkalainen@thl.fi; 5 Institute of Molecular Medicine, Faculty of Medicine. University of Lisbon, 649-028 Lisbon, Portugal

**Keywords:** organic dust, occupational exposure, cytotoxic effects, inflammatory effects, in vitro

## Abstract

Organic dust and related microbial exposures are the main inducers of several respiratory symptoms. Occupational exposure to organic dust is very common and has been reported in diverse settings. In vitro tests using relevant cell cultures can be very useful for characterizing the toxicity of complex mixtures present in the air of occupational environments such as organic dust. In this study, the cell viability and the inflammatory response, as measured by the production of pro-inflammatory cytokines tumor necrosis factor-α (TNFα) and interleukin-1 β (IL-1β), were determined in human macrophages derived from THP-1 monocytic cells. These cells were exposed to air samples from five occupational settings known to possess high levels of contamination of organic dust: poultry and swine feed industries, waste sorting, poultry production and slaughterhouses. Additionally, fungi and particle contamination of those settings was studied to better characterize the organic dust composition. All air samples collected from the assessed workplaces caused both cytotoxic and pro-inflammatory effects. The highest responses were observed in the feed industry, particularly in swine feed production. This study emphasizes the importance of measuring the organic dust/mixture effects in occupational settings and suggests that differences in the organic dust content may result in differences in health effects for exposed workers.

## 1. Introduction

Organic dust is usually defined as an airborne mixture of viable and non-viable microorganisms (bacteria, fungi, viruses, protozoa), their metabolites (endotoxins, glucans, mycotoxins, peptidoglycans, enzymes etc.) and solid particles of vegetable and animal origin (allergens, including pollens, vegetal fibers, epidermis etc.) [1,2]. Organic dust and related microbial exposures are the main inducers of several respiratory symptoms, such as decline in lung function, asthma, chronic bronchitis, bronchial hyper-responsiveness, wheeze, and cough [1,3,4,5,6,7,8]. 

Occupational exposure to organic dust is very common and has been reported in several diverse settings. The most commonly reported settings are those related with animal handling and feed production but also farming [5,9,10,11,12]. Bakeries, waste and water management and greenhouses are other types of settings also mentioned in the literature [13,14,15,16,17,18,19]. Recently published work showed that in slaughterhouses there is also occupational exposure to organic dust [12,20]. However, despite an apparent adaptation response of workers repeatedly exposed to organic dust [21,22,23], they still experience a high prevalence of respiratory disease and a significant decline in lung function [24,25]. Apart from their allergic and infectious properties, bacteria and fungi can induce inflammatory responses via inhalation of endotoxin or ß-glucans [26,27]. Furthermore, the respiratory symptoms observed in bioaerosol-exposed workers are thought to be mainly caused by non-allergic inflammatory reactions [28]. Mononuclear phagocytes, primarily monocytes and macrophages, are the key cells that initially respond to exposure of inhaled organic dust by rapidly stimulating secretion of tumor necrosis factor (TNF) [29]. Monocyte/macrophage-derived inflammatory mediators can induce pyrexia, neutrophil recruitment, and activation of airway epithelial cells, and cause direct bronchial hyper-reactivity [10,30,31]. 

A possible methodological approach to characterize the toxicity of the complex mixtures present in the air of occupational environments is based on biological testing, which produces a global response to the complex mixtures of chemicals and biological agents without any prior knowledge of the mixture composition or its properties [32]. In such a context, in vitro testing using relevant cell cultures might provide useful information on health effects of co-exposure to multiple stressors. 

The objective of this study was to evaluate the relation between the presence of organic dust in different occupational settings and the occurrence of proinflammatory effects. For this purpose, inflammatory response and cell viability were investigated in vitro in human macrophages exposed to air samples of five different occupational settings characterized by high exposure to organic dust, namely: poultry and swine feed industries, waste sorting, poultry production and slaughterhouses. Additionally, contamination by fungi and particles in those settings was studied to better characterize the organic dust composition.

## 2. Materials and Methods

### 2.1. Occupational Environments

Five different occupational environments, all located in the Lisbon region, were assessed between November 2015 and January 2016 during normal working days. The selected settings were: one poultry feed industry (PFI), one swine feed industry (SFI), one waste sorting plant (WSP), one poultry pavilion (PP), and one slaughterhouse (S). In both feed industries, the raw materials arriving by train or by trucks are cleaned and stored in silos. In a batch process, the raw materials are ground and mixed with fats, molasses, and additives such as vitamins and minerals. The mixture is then usually pressed into pellets and stored in silos again. The animal feed is either packed and shipped in sacks or shipped in bulk trucks. The process is highly mechanized and operated mostly from a central control room. Exposure of workers therefore mainly takes place during unloading, cleaning, maintenance and during manual mixing of some specific components. The units work 5 days a week with a daily regimen of two 8-h shifts. The WSP has a maximum capacity of 90,500 tons/year of waste. This plant functions 5 days/week in a daily regimen of two 8-h shifts. The PP selected is dedicated to broiler chicken production (density of 15 broiler chicken/m^2^), where birds are bred to reach slaughter weight as rapidly as possible. One-day-old chicks are transferred from hatcheries to the growing farms, where they are housed in single-story sheds. The litter was composed of rice hulls and had 33 days of use. Poultry staff monitor the condition of the birds daily, adjust feed and water equipment as necessary, and administer vaccines. The S has the capacity for slaughtering 150 tons/day of swine and bovine animals. The sampling sites selected for each of these settings were chosen based on the large amount of time spent by the workers on those places during their occupational activity (Table 1). Of note, none of the workers used respiratory protection devices in any of the evaluated workplaces.

### 2.2. Fungal Burden Assessment

#### 2.2.1. Samples Collection

Two air sampling methods were applied to each sample—the impinger method and the use of filters. All air samples collected by the impinger method were obtained with the impinger Coriolis μ air sampler (Bertin Technologies, Montigny-le-Bretonneux, France). Samples of 300 L were collected at 300 L/min airflow rate into 10 mL of sterile phosphate-buffered saline (PBS) with 0.05% Triton X-100. Duplicates were collected in each sampling site. In parallel, the aerosol monitor (DustTrak II model 8532, TSI^®^, Minnesota, MN, USA) was used to assess viable microbiological material below 2.5 µm in size. For that purpose, a PM2.5-µm sampling head and a PVC filter with a diameter of 37 mm were applied to the equipment. In each location, and after performing a blank sample, 30-min (2 L/min) flow rate sampling was performed. The selected period of time was representative of the task intended to be assessed.

#### 2.2.2. Sample Preparation and Analysis

● Conventional methodologies

Samples were prepared for analysis by spreading 150 µL of the previously described suspension from the collection liquid onto malt extract agar (2%) with chloramphenicol (0.05 g/L). In the case of filters, they were immersed in 300 mL of sterilized distilled water, followed by agitation for 30 min at 100 rpm, and then 150 µL were spread onto malt extract agar (2%) with chloramphenicol (0.05 g/L). These samples were incubated at 27 °C for 5–7 days. After laboratory processing and incubation of the collected samples, quantitative (using colony-forming units, CFU/m^3^ and CFU/m^2^) and qualitative results were obtained, with identification of all the isolated fungal species. For species identification, microscopic mounts were performed using tease mount or Scotch tape mount and lactophenol cotton blue mount procedures. Morphological identification was achieved through macro and microscopic characteristics, as noted by De Hoog et al. [33].

● Molecular methodologies

Molecular methods were applied to collected air samples in order to detect fungi, as a complement to conventional methods. This combined approach was performed to overcome some limitations of the culture-based methods and whenever specific species/strains needed to be detected due to their toxigenic potential. Briefly, five milliliters of the collection liquid were centrifuged at 2500× *g* for 10 min, the supernatant was removed and DNA was extracted using the ZR Fungal/Bacterial DNA MiniPrep Kit (Zymo Research) according to the manufacturer’s recommendations. Molecular identification of *Aspergillus* sections *Flavi* (toxigenic strains), *Fumigati* and *Circumdati* (Table 2) was achieved by real time quantitative PCR (qPCR) using the Rotor-Gene 6000 qPCR Detection System (Corbett). Reactions included 1× iQ Supermix (Bio-Rad), 0.5 μM of each primer (Table 2), and 0.375 μM of TaqMan probe in a total volume of 20 μL. Amplification followed a three-step PCR: 40 cycles with denaturation at 95 °C for 30 s, annealing at 52 °C for 30 s, and extension at 72 °C for 30 s. A non-template control was used in every PCR reaction. As positive controls, we used DNA extracted from reference strains from the Mycology Laboratory from the National Institute of Health Doutor Ricardo Jorge (INSA).

### 2.3. Particles Assessment

Particle measurements were performed with an aerosol monitor (DustTrak II model 8532, TSI^®^) aiming to assess particle masses of 2.5 µm in size. For that purpose, a PM2.5-µm sampling head was applied to the equipment. Each measurement was done over 15 min in each workplace and during task performance. In the case of the poultry pavilion and the slaughterhouse it was not possible to perform particle assessment due to unavailability of the equipment.

### 2.4. Cytotoxic and Inflammatory Assessment

For the toxicological characterization, cytotoxicity and inflammatory (IL-1β and TNF-α) responses were analyzed using methods described earlier [34]. Macrophages were chosen because they play a role in triggering the inflammatory response through secretion of cytokines. Human monocytic THP-1 cells (American Type Culture Collection, Manassas, VA, USA) were grown in RPMI medium supplemented with 10% fetal bovine serum, 2 mM L-glutamine, 0.05 mM 2-mercaptoethanol, 100 U/mL penicillin and 100 µg/mL streptomycin (all from Gibco, Life Technologies, Carlsbad, CA, USA) at 37 °C in a humidified atmosphere of 5% CO_2_ in air. The cells were differentiated into macrophages with phorbol 12-myristate 13-acetate (PMA, Sigma-Aldrich, St. Louis, MO, USA) after which the cells turned adherent. After 48 h, the differentiation medium was replaced with exposure medium. Exposure medium contained samples in PBS (collected by impinger method) in dilutions of 1:20 and 1:50, or PBS vehicle only. For the cytotoxicity assessment, cells were grown on 96-well plates, 65,000 cells/well (cell culture plastics from Nunc, Roskilde, Denmark). Cell viability was determined by colorimetric assay using Cell Proliferation Reagent WST-1 (Roche, Mannheim, Germany). Exposure medium was removed after 18 h treatment and WST-1 reagent was added to wells. Cells were incubated for 1 h. After shaking the cell plate for 1 min, the absorbance of the samples was measured using a plate reader at 450 nm (EnSpire, Perkin Elmer, Waltham, MA, USA). For the cytokine analysis, cells were grown on 6-well plates, 1.6 million cells/well. After 18 h of exposure, the medium was collected for analysis. The secretion of proinflammatory cytokines TNFα and IL-1β into the cell culture medium was determined using the commercial kits Human TNFα and IL-1β DuoSet ELISA Development System combined with Ancillary Reagent Kit (R&D System, Minneapolis, MN, USA) according to the manufacturer’s instructions. Table 3 summarizes the assays performed for each sampling site at all evaluated settings.

### 2.5. Data Analysis

The data analysis was performed and descriptive statistics was applied, using either frequency, median or graphical representations in accordance with the nature of the data. In addition, to test whether there were significant differences between settings, the Kruskal–Wallis test was used. The cell viability data was analyzed by ANOVA followed by the Mann–Whitney test. Statistical software SPSS V21 was applied for statistical analysis. The results were considered significant at a 5% significance level.

## 3. Results

### 3.1. Fungal Burden

As expected, higher fungal load (564 out of 712 isolates) and a wider diversity of fungal species (higher number of different species in all settings with exception of the slaughterhouse since both methods presented only *Chrysonilia sitophila*: PFI 4 different species out of 6; SFI 4 out of 7; WSP 11 out of 12; PP 4 out of 6) were found using impinger method since this method collected all viable fungal material, whereas the filter method collected only fungal material with a particle size smaller than 2.5 µm. 

#### 3.1.1. Fungal Load

**Figure 1:**
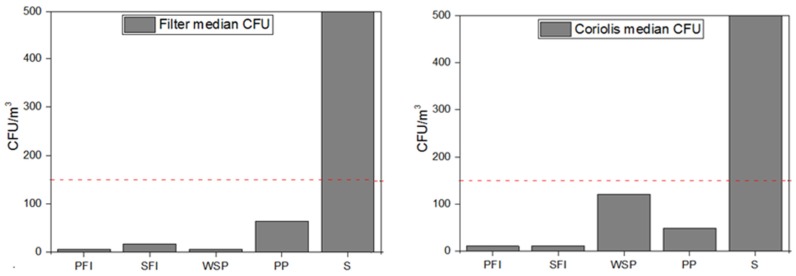
Fungal load distribution in the five occupational environments with both sampling methods applied (Filter and Impinger Coriolis), respectively. Dashed line represents reference limits suggested by World Health Organization (WHO). Countless colonies were counted as 500 colony-forming units (CFU).

The fungal load in the air of the assessed occupational environments presented different ranges between impinger and filter methods (Figure 1). The waste sorting plant had one sampling site that exceeded the limits of the guideline proposed by World Health Organization (WHO) (maximum value of 150 CFU/m^3^) [34]. We should also consider the same situation in one sampling site for the SFI through filter method, one sampling site in the PFI by impinger method and in six sampling sites (three for each method) in the S due to fungi with fast growing rates (with overloaded plates). No fungal growth was obtained in one sample using impinger method and three samples using filter method.

#### 3.1.2. Fungal Identification

In the six units assessed in the PFI, a total of 48 isolates were obtained through the impinger method. The filter assay led to the detection of 12 isolates. *Chrysonilia sitophila* overgrowth in the pre-mixing was also observed (Table 4). In the SFI, four different fungal species were detected in indoor air by impinger method in a total of 54 isolates. In the filter assay, 34 isolates were obtained (not considering the *C. sitophila* overgrowth in the warehouse) (Table 4). The impinger method enabled the identification of 413 fungal isolates, from twelve different genera/species in the WSP. In the filter assay 38 isolates from *Penicillium* sp. were obtained (Table 4). In the PP, 49 isolates of four different genera/species were identified through the impinger method and 64 isolates through the filter assay (Table 4). In the S, both the impinger method and the filter assay were able to identify *C. sitophila* overgrowth. It was not possible to identify any countable colonies of other fungal species (Table 4).

#### 3.1.3. Fungal Detection

Toxigenic strains from *Aspergillus* sections *Flavi* and *Circumdati* were not amplified by qPCR. However, *Aspergillus* section *Fumigati* DNA was amplified in most settings, although not to a greater extent as cycle threshold (CT) values obtained are quite high. In the PFI, *Aspergillus* section *Fumigati* was amplified in the manual mixing (CT 37.68) and granulator (CT 38.46) settings whereas in the SFI, this complex was detected in the bagging line (CT 37.94) and in the warehouse of the final product (CT 37.85). The same *Aspergillus* section was also amplified in the PP (CT 37.39) as well as in the S in swine gutting (CT 35). Finally, in the WSP, this section was amplified in two workstations, namely: alveoli (CT 36.97) and waste without sorting (CT 38.11) workstations. In addition, qPCR analysis successfully amplified DNA from the *Aspergillus* section *Fumigati* in seven sampling sites where the presence of this fungal species had not been identified by conventional methods. Of note, considering that air samples had the same initial volume, it is very likely that samples with lower cycle threshold values exhibit higher levels of *Aspergillus* section *Fumigati*.

### 3.2. Particles

Due to unavailability of the equipment, data from particle contamination was obtained only in three occupational settings. The PFI showed higher contamination, probably because there are no risk management measures, such as local exhaust ventilation and/or general mechanical ventilation. Only in the WSP was there this kind of ventilation resource, in the sorting cabinets above the sorting belt (Table 4).

Statistically significant differences in particulate matter concentration ($\chi_{KW}^{2}$ (2) = 35,342, *p* = 0.000) were detected between the three assessed settings (PFI, SFI and WSP). In addition, statistically significant differences between the setting poultry feed productions and the other two settings (*p* < 0.05) were obtained through the Kruskal–Wallis multiple comparison test. The PFI presented higher particulate matter concentration values, and the WSP and SFI showed similar values.

### 3.3. Cytotoxicity and Pro-Inflammatory Effects

**Figure 2:**
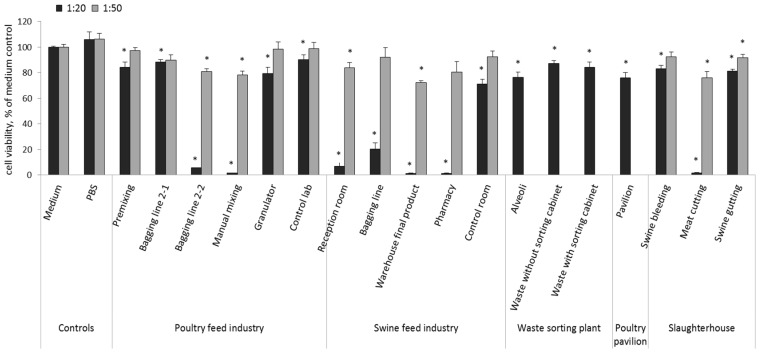
Cell viability of macrophages derived from THP-1 cells after treatment with air samples collected from the five occupational settings as calculated by % of medium control. Columns represent mean values ± standard error SE (*n* = 4) in two independent experiments using dilutions 1:20 and 1:50. Dilution 1:50 is missing from the WSP and PP settings, since 1:20 dilution was not highly cytotoxic to cells in the first experiment. Statistical significant differences (*p* < 0.05) between samples and medium control are marked with asterisks (*). PBS: phosphate-buffered saline.

The toxicological characterization of the analyzed samples showed a concentration-dependent cytotoxic effect of the measured endpoints. The highest cytotoxic response to the air samples of different workplaces were found in the SFI (final product warehouse, pharmacy, reception, bagging line), PFI (bagging line 2-2, manual mixing), and S (meat cutting) (Figure 2). 

**Figure 3:**
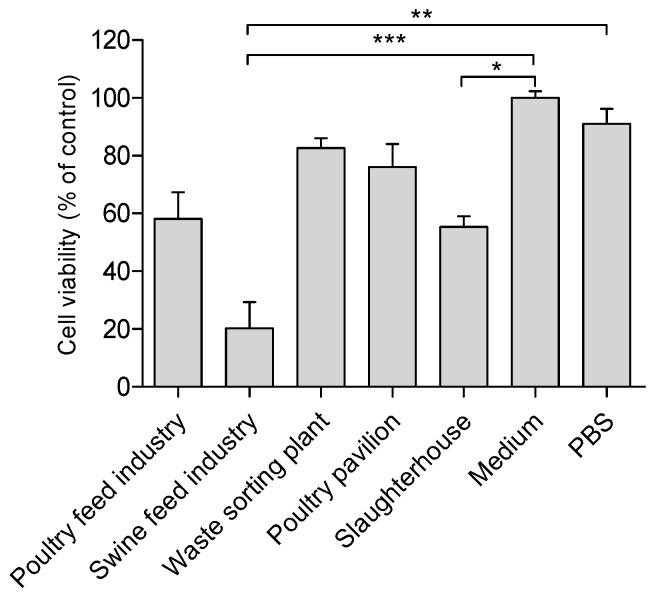
Relative cell viability of THP-1 cell line. Columns represent mean values ± SEM (*n* = 4) for dilution 1:20 of air samples from five occupational settings. Statistical differences between the five occupational settings and control groups (medium and PBS) are reported as *** *p* < 0.001, ** *p* < 0.01, * *p* < 0.05. Cell viability (% of control) = (A) test/(A) control × 100.

When calculating the mean cell viability after exposure to air samples from the five different occupational settings it was found that for the poultry feed industry, waste sorting plant, poultry pavilion and in the slaughterhouse settings, about 60% of cells were alive after exposure to air samples at dilution 1:20, whereas in the swine feed industry only about 20% of cell were alive after similar exposure (Figure 3). Statistically significant differences were detected between the tested groups (five settings and two controls) through the Kruskal–Wallis test ($\chi_{KW}^{2}$ (6) = 36.02, *p* < 0.00001). 

**Figure 4:**
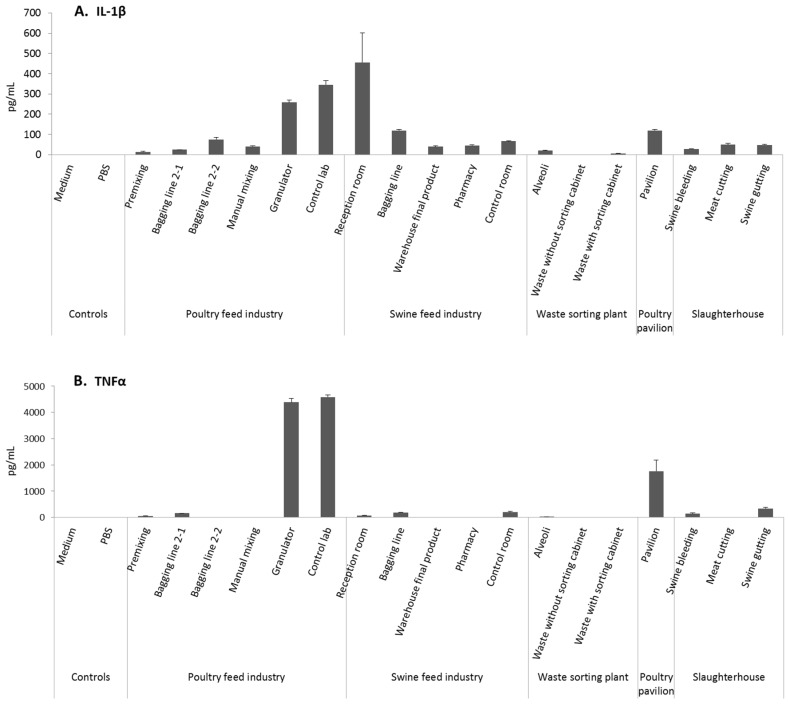
IL-1β (A) and TNFα (B) responses after treatment of macrophages derived from THP-1 monocytic cells with samples collected from the different occupational settings using dilution 1:20. The controls contained medium or PBS in the place of samples. Columns represent mean ± SE of two replicates from one representative experiment out of two independent experiments.

The swine feed industry, poultry feed industry, and poultry pavilion were the settings with higher pro-inflammatory-type responses, as a strong release of pro-inflammatory mediators (IL-1β, TNFα) was detected following in vitro incubation of human macrophages with air samples from these settings (Figure 4). The highest levels of IL-1β were observed following exposure to samples from the SFI (reception room), and from the PFI (granulator and laboratory). In addition, the highest levels of TNFα were detected with air samples from the poultry feed industry (granulator and laboratory), and from the PP (Figure 4). Notably, the basic levels of these cytokines were very low, with any stimulating factors often below the detection limits of the kits used in measurements. Therefore the unexposed controls gave no measurable values and the statistical comparison against them was impossible.

## 4. Discussion

In the present study, we evaluated the cytotoxic and pro-inflammatory effects induced in vitro by air samples collected from workplaces, (feed industries, poultry production, slaughterhouse and waste sorting plant), where workers are exposed to organic dust. Results obtained allow the understanding that for the different settings, while all involve high levels of organic exposure, there are different impacts on the health of exposed workers. It seems that the workers in the feed industry, particularly in swine feed production, might have the highest inflammatory responses due to organic dust exposure.

Macrophages were chosen for the in vitro assessment because they are known to be responsible for first-line protection and also for triggering the inflammatory response via secretion of signaling molecules. Human monocytic THP-1 cells have become one of most widely used cell lines to investigate immune responses. Our goal was to study the real exposure scenarios, which are characterized by complex mixtures of individual microbial and chemical agents. Numerous studies [35,36,37], have demonstrated that there may be a close interaction between different agents and that these interactions may modulate the effect of the single pollution component [38,39]. Moreover, several authors have stated that, instead of investigating the unique effects of specific pollutants [40,41,42,43] it might be more reasonable to assess the harmful effects of mixture of pollutants. Nevertheless, this approach has also some limitations. It was not possible to identify and quantify all contaminants present in the dust samples and therefore no direct link could be made between a specific contaminant and the biological responses analyzed. It was also not possible to relate the effects with a specific dose of each contaminant; however, previous studies have supported the idea of a dose-dependent effect. In particular, a study by Miller et al. (2010) [44] confirmed the inflammatory nature of fungal metabolites/toxins and their contribution to the development of non-allergenic respiratory health effects and the time- and dose-dependency of the observed effects. The same trend has also been found for endotoxins in several studies [8,15,45,46,47]. A previous study by Mackiewicz et al. (2015) [48] proved also a significant relationship between the concentrations of total microorganisms in the air of agricultural settings and the occurrence of respiratory disorders in the exposed workers. In our study, the use of two different sampling methods allowed us to obtain two insights into the assessments performed. On one hand, with the impinger method, higher fungal load and a wider diversity of fungal species were found since this method collected all viable fungal material. On the other hand, with the filter method, it was possible to obtain information regarding fungal occupational exposure, but also about viable fungal particle dimensions. This is an important achievement concerning risk assessment since it is possible to obtain information regarding not only the characterization of fungal contamination, but also the size of dust particles, which is important in terms of transport function and because size exerts an influence on the health effects of fungi [49,50,51,52]. Finally, lifestyle, together with environmental and/or occupational exposures, can have an additive or a synergistic impact [53]. In this study, all air samples collected promoted cytotoxic and pro-inflammatory responses. Of note, the highest responses in both endpoints were observed in the feed industry, particularly in the swine feed industry. The differences in responses between settings can be explained by the different organic dust composition in distinct workplaces, due to the type of material that is handled, the raw materials that are used, the processes involved, and the environment of workplaces. In the case of poultry feed, particle mass was much greater in comparison to that found in waste sorting and the swine feed industry. This might explain the high values in cytotoxicity and inflammatory effects since particles have an oxidative potential to induce the generation of cellular reactive oxygen species related with inflammatory outcomes [54]. It should also be noted that the air samples were collected into a PBS containing Triton X-100, which itself may influence cytotoxicity and cytokine production. However, the concentration of Triton X-100 in our exposure medium was, at its highest, only 0.0025% and that level had no effect on cell viability and proinflammatory responses. 

Of note, organic dust may serve as a favorable medium for the persistence of numerous species of bacteria and microscopic fungi, which, in addition, may release metabolites that exert different health effects in workers [55]. Taking into consideration the most prevalent fungi detected in swine and poultry feed industries, and in the poultry pavilion (Table 4) (*Acremonium* sp., *Penicillium* sp., *Aureobasidium* sp., *Rhizopus* sp., *Cladosporium* sp., etc.), our results suggest that these fungal species, alone or most likely in combination, might have contributed to some extent to the observed cytotoxicity and pro-inflammatory phenomena. In addition, the identification of *Aspergillus* section *Fumigati* in these five settings might also partially explain the induced pro-inflammatory effects, since this clinically important species is responsible for the abundant production of gliotoxin, a mycotoxin with a diverse array of biologic effects on the immune system [12]. However, the observed pro-inflammatory effect is most likely related to the complex composition of organic dust, and therefore the results must be interpreted in association with particulate matter load and other components of the organic dust. Moreover in a recent review, Tsapko et al. (2011) [56] found a direct positive correlation between the concentrations of dust and microorganisms in the air of the working zone. Ellen et al. (2000) [57] observed the same trend in poultry farms with respect to particulate matter size distribution. Endotoxins were not measured but, as previously reported, they are probably present in all the occupational settings analyzed [9,13,58,59]. Moreover, endotoxins have been reported as being responsible for inflammatory responses [60,61,62,63]. The (1–3)-b-d-glucan from fungi is a common component of organic dust that might cause inflammatory airway reactions and also affect the immune system when inhaled [64,65]. The (1–3)-b-d-glucans are present in the cell wall of filamentous fungi, yeasts and some bacteria [66]. There is an increasing evidence that (1–3)-b-d glucans are potent inducers of inflammation, causing non-specific inflammatory reactions [26,67,68], as well as being as modulators of the immune system, being thus potentially responsible for the main inflammatory effect of fungi. The biological activities of (1–3)-b-d glucan include host-mediated antitumor activity, adjuvant effects, activation of neutrophils, eosinophils, macrophages and complement [69]. Besides fungi, cereals commonly used in feed production, such as oats and barley, also produce (1–3)-b-d-glucans [70], which may partly explain the higher inflammatory response observed in air samples collected from the two assessed feed settings. At the molecular level, future studies to be developed, based on fungi calibration curves, will allow for quantification of fungal load based on DNA amplification and, therefore increased ease of correlation with the data obtained. Furthermore, it is important to note that, in addition to particles and fungi [71], mycotoxins are commonly reported as feed contaminants involved in severe health problems in animals and also in humans [72,73,74,75,76]. The most common mycotoxins in the swine feed are aflatoxins (AF), deoxynivalenol (DON), zearalenone (ZEA), ochratoxin (OTA) and fumonisins [77]. Previous studies in poultry and swine production, waste management and slaughterhouses demonstrated occupational exposure of workers to aflatoxin B1 (AFB1), with the inhalation route probably being the most significant exposure route in almost all settings [20,78,79]. Although mycotoxin content was not determined in the present study, it is very likely that mycotoxins were present in the collected samples. Inhalation exposure to mycotoxins results in a variety of respiratory health events [80], which can be worsened by the presence of other pollutants, such as particles [81]. Capasso et al. (2015) [81] reported for the first time the effects of co-exposure of DON and PM10 on cell death, interleukin release and cell cycle alterations in human lung epithelial cells. Low doses of particles and DON alone had scarce toxic potency per se, while together they were able to trigger cytotoxicity and sustain inflammatory effects [81]. Therefore, it is possible that co-exposure to several mycotoxins and particles in the swine feed industry led to the high inflammatory responses observed in this study. The scarce use of respiratory protection devices by workers in all evaluated workplaces can also contribute to the negative effects on workers’ health. Thus, wearing respiratory protection, particularly in the tasks with higher exposure to dust, can make an important contribution to reducing exposure.

Knowledge about this kind of interactions present in the workplaces is a key aspect regarding the use of occupational exposure limits (OELs) since in most of the workplaces the workers are exposed to mixtures and not to a single agent to which an OEL has been defined [82]. This allows us to better understand the limitations of OELs in those cases where health effects are being observed even when exposure values are below the OEL. Moreover, smoking, as well as pre-existing respiratory conditions (atopy, asthma), may modify the development of bioaerosol-related symptoms, due to a synergistic effect. This study emphasizes the importance of considering that the real exposure scenario in occupational settings is characterized normally by an exposure to a complex mixture. In fact, the health effects are probably associated more with the exposure to the mixture than to each contaminant per se. Therefore, in order to determine the potential health risk, it is important to analyze the mixture of effects in different occupational settings where workers are exposed to organic dust. Additionally, each occupational setting has differences in the organic dust composition and this can result in different health effects among exposed workers. Our findings suggest that the workers in the feed industry, especially in swine feed production, might have the highest inflammatory responses due to organic dust exposure when comparing all occupational settings assessed in this study.

## 5. Conclusions

This study corroborates the importance of considering exposure to complex mixtures in occupational settings, which is a commonly occurring event. More studies are needed to drive robust conclusions on the effect of combined and multiple exposure to complex mixtures on public health in general and in particular, in occupational settings. Indeed, of several studies currently addressing the health effects associated with simultaneous exposure to several environmental contaminants, only very few address the quantitative estimates of the combined effect on health. 

## Figures and Tables

**Table 1:** Sampling sites selected from each occupational environment. Each sampling site corresponds to the workplaces where the workers spend more time.

Poultry Feed Industry (PFI)	Swine Feed Industry (SFI)	Waste Sorting Plant (WSP)	Poultry Pavilion (PP)	Slaughterhouse (S)
Premixing	Reception room	Alveoli (waste discharging area)	Pavilion	Swine bleeding
Bagging line 2-1	Bagging line	Waste without sorting cabinet	-	Meat cutting
Bagging line 2-2	Final product warehouse	Waste with sorting cabinet	-	Swine gutting
Manual mixing	Pharmacy	-	-	-
Granulator	Control room	-	-	-
Control lab	-	-	-	-

**Table 2:** Sequence of primers and TaqMan probes used for real time PCR.

*Aspergillus* Sections	Sequence
*Aspergillus* section *Flavi* (toxigenic strains)	
Primer Forward	5′-GTCCAAGCAACAGGCCAAGT-3′
Primer Reverse	5′-TCGTGCATGTTGGTGATGGT-3′
Probe	5′-TGTCTTGATCGGCGCCCG-3′
*Aspergillus* section *Fumigati*	
Primer Forward	5′-CGCGTCCGGTCCTCG-3′
Primer Reverse	5′-TTAGAAAAATAAAGTTGGGTGTCGG-3′
Probe	5′-TGTCACCTGCTCTGTAGGCCCG-3′
*Aspergillus* section *Circumdati*	
Primer Forward	5′-CGGGTCTAATGCAGCTCCAA-3′
Primer Reverse	5′-CGGGCACCAATCCTTTCA-3′
Probe	5′-CGTCAATAAGCGCTTTT-3′

**Table 3:** Number of samples collected and assessments performed.

Occupational Environments	Conventional Methods		Molecular Biology	Particulate Matter	In Vitro Toxicological Assessment
	Impinger Method	Filter Method			
**Poultry feed industry (PFI)**	5	5	5	5	5
**Swine feed industry (SFI)**	6	3	6	3	6
**Waste sorting plant (WSP)**	3	3	3	3	3
**Poultry pavilion (PP)**	1	1	1	Not assessed	1
**Slaughterhouse (S)**	3	3	3	Not assessed	3
**Total of samples**	18	15	15	11	18

**Table 4:** Particle concentrations measured in three different occupational settings, with mass average (mg/m^3^) for each workplace, mass average, minimum, maximum, and standard deviation for each setting, and Kruskal–Wallis test results. SD: standard deviation.

Settings	Workplace	Mass Average (mg/m^3^)	mg/m^3^	Kruskal–Wallis Test Results				
				*n*	Mean Rank	$\chi_{KW}^{2}$	df	*p*
Poultry feed industry (PFI)	Bagging line (5 kg)	0.181	Mass average 0.098 (min.–max.) (0.028–0.198) SD 0.061	90	141,02	35,342	2	2.1 × 10^−8^
	Premixing control room	0.074						
	Control lab	0.038						
Swine feed industry (SFI)	Reception room	0.113	Mass average 0.054 (min.–max.) (0.007–0.143) SD 0.042	150	149,81			
	Bagging line	0.053						
	Warehouse final product	0.080						
	Pharmacy	0.014						
	Control room	0.010						
Waste sorting plant (WSP)	Alveoli	0.049	Mass average 0.049 (min.–max.) (0.036–0.062) SD 0.007	90	216,13			
	Pre Screening	0.044						
	Screening	0.053						
